# Supplementary material for: A GABARAP−PtdIns3K-C1 positive feedback loop at the heart of the phagophore nucleation
Source: Nat Commun. 2026 Jul 30;17:9473. doi: 10.1038/s41467-026-76135-w (PMC13542195; doi:10.1038/s41467-026-76135-w)
Supplement: Supplementary file 1 [file 41467_2026_76135_MOESM1_ESM.pdf]

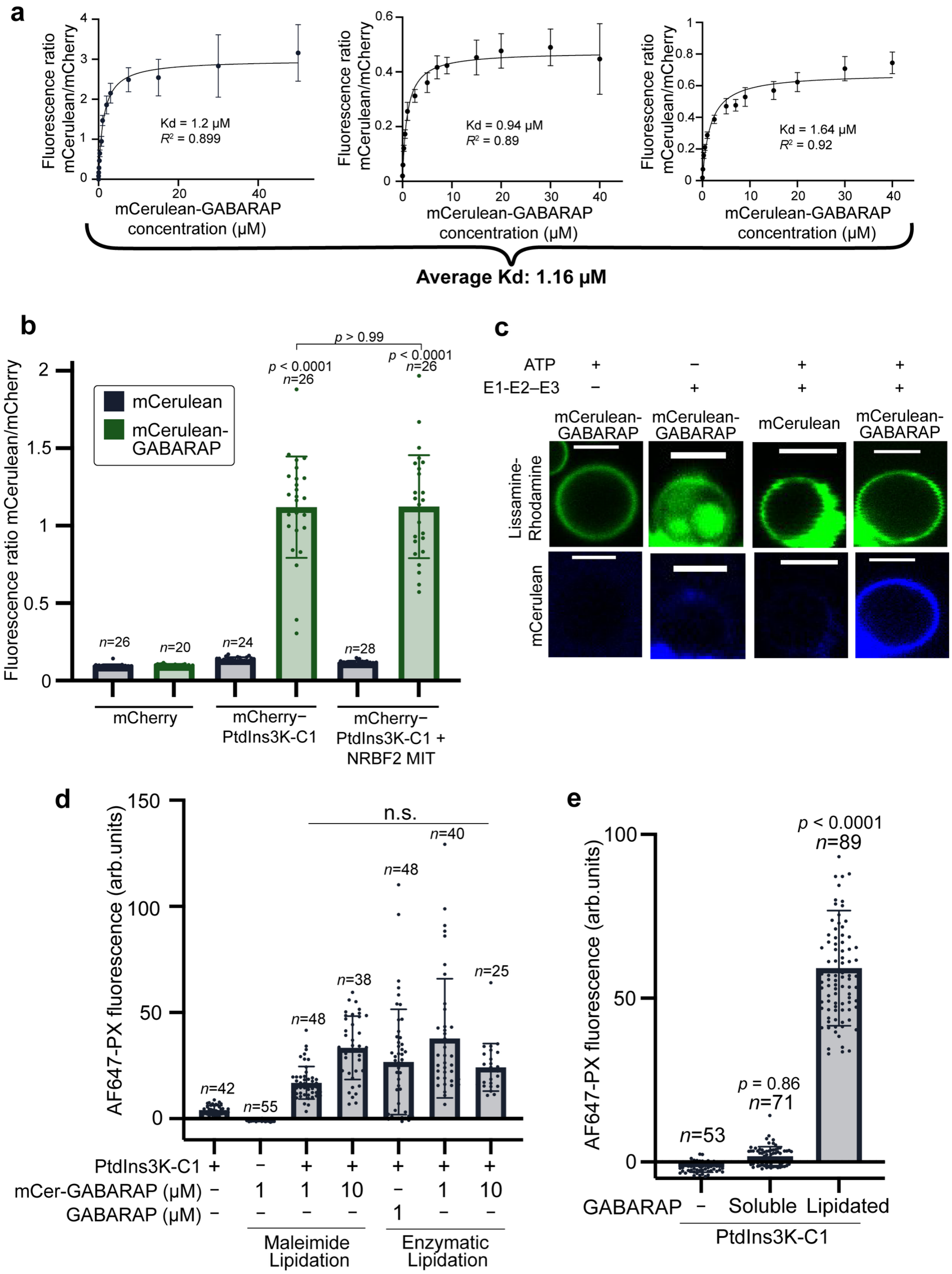

Supplementary Figure 1. Controls for the binding and the activation of PtdIns3K-C1 with GABARAP. a Affinity of PtdIns3K-C1 for GABARAP. Replicates for the experiment shown in Fig. 2c. The plots show the average of at least 29 beads ± *SD*. A Kd and associated R^2 were determined by a non-linear fit. **b** mCherry, mCerulean and NRBF2 MIT do not affect the binding between PtdIns3K-C1 and GABARAP. mCherry or mCherry–PtdIns3K-C1 were immobilized to beads as presented in Fig. 2a, and incubated with mCerulean or mCerulean-GABARAP, in the presence or the absence of the NRBF2 MIT domain. **c** The lipidation of mCerulean-GABARAP to GUVs is not caused by the mCerulean protein, and needs ATP and the lipidation enzymes E1-E2–E3. Representative confocal micrographs of GUVs, in the Lissamine-Rhodamine (lipids) and mCerulean channels. Scale bars: 5 µm. **d** Comparison of the enzymatic and maleimide-based GABARAP lipidations for PtdIns3K-C1 activity. mCerulean (mCer)-GABARAP was conjugated to GUVs through maleimide reaction or the enzymatic lipidation cascade, then

incubated with PtdIns3K-C1 and AF647-PX to follow PtdIns3P production in an endpoint. A control with GABARAP instead of mCer-GABARAP shows the mCerulean does not affect PtdIns3K-C1 activation. Average of the AF647-PX fluorescence intensity in arbitrary units for *n* GUVs ± *SD.* Averages were compared to the sample with PtdIns3K-C1 but without GABARAP with a one-way ANOVA with Tukey's correction for multitesting (n.s.: $p > 0.05$). **e** Soluble GABARAP does not activate PtdIns3K-C1. GUV-based endpoint PtdIns3K-C1 activity assay using GUVs with soluble or maleimide-lipidated GABARAP. Average of the AF647-PX fluorescence intensity for *n* GUVs ± *SD.* Means were compared to the PtdIns3K-C1 only control with a one-way ANOVA with Tukey's correction for multitesting.

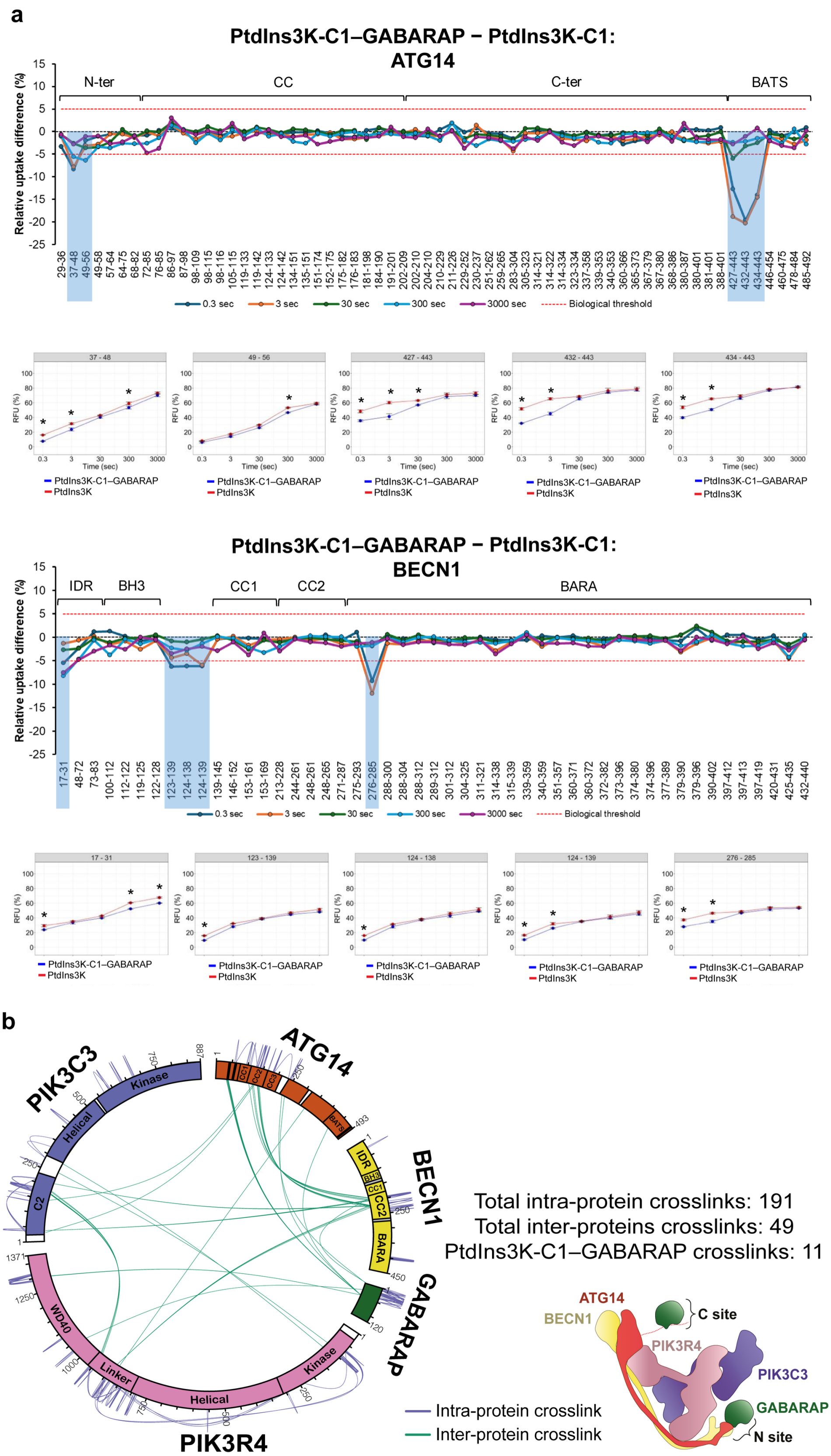

Supplementary Figure 2. Identification of two GABARAP-binding sites on PtdIns3K-C1 by a combination of structural approaches. a HDX-MS difference plots for the ATG14 and BECN1 subunits. Peptides showing significant differences (PtdIns3K-C1–GABRAP – PtdIns3K-C1) are framed in blue (protection) and their corresponding uptake plots are displayed. Stars indicate timepoints showing significant differences. No significant differences have been identified for the PIK3R4 and PIK3C3 subunits. **b** Crosslinking-MS maps on PtdIns3K-C1 subunits. Identified crosslinked peptides between PtdIns3K-C1 and soluble GABARAP are mapped on the different protein domains. Numbers of intra-protein crosslinks (blue) and inter-protein crosslinks (green) are indicated.

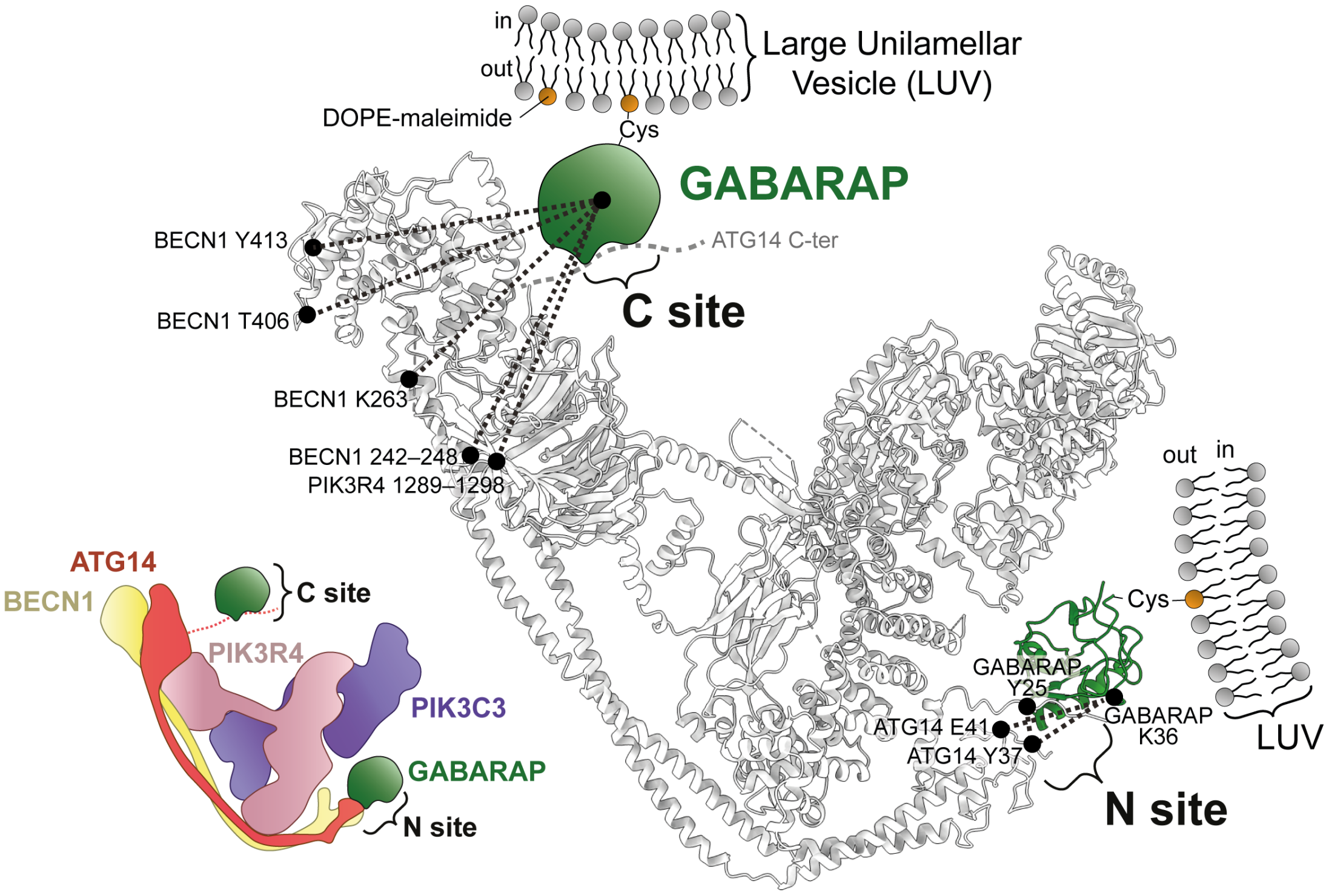

C site

XL-BECN1	XL-GABARAP	Cα-Cα distance (Å)
E242	K46	N/A
D244	K46	N/A
D245	K46	N/A
E246	K46	N/A
K248	L44	N/A
K248	D45	N/A
K263	E17	N/A
T406	D27	N/A
Y413	Y25	N/A

XL-PIK3R4	XL-GABARAP	Cα-Cα distance (Å)
S1289	K46	N/A
Y1291	Y25	N/A
Y1291	K46	N/A
K1293	I41	N/A
T1298	I41	N/A

N site

XL-ATG14	XL-GABARAP	Cα-Cα distance (Å)
Y37	Y25	11.1
Y37	K46	22.7
E41	K46	23.2

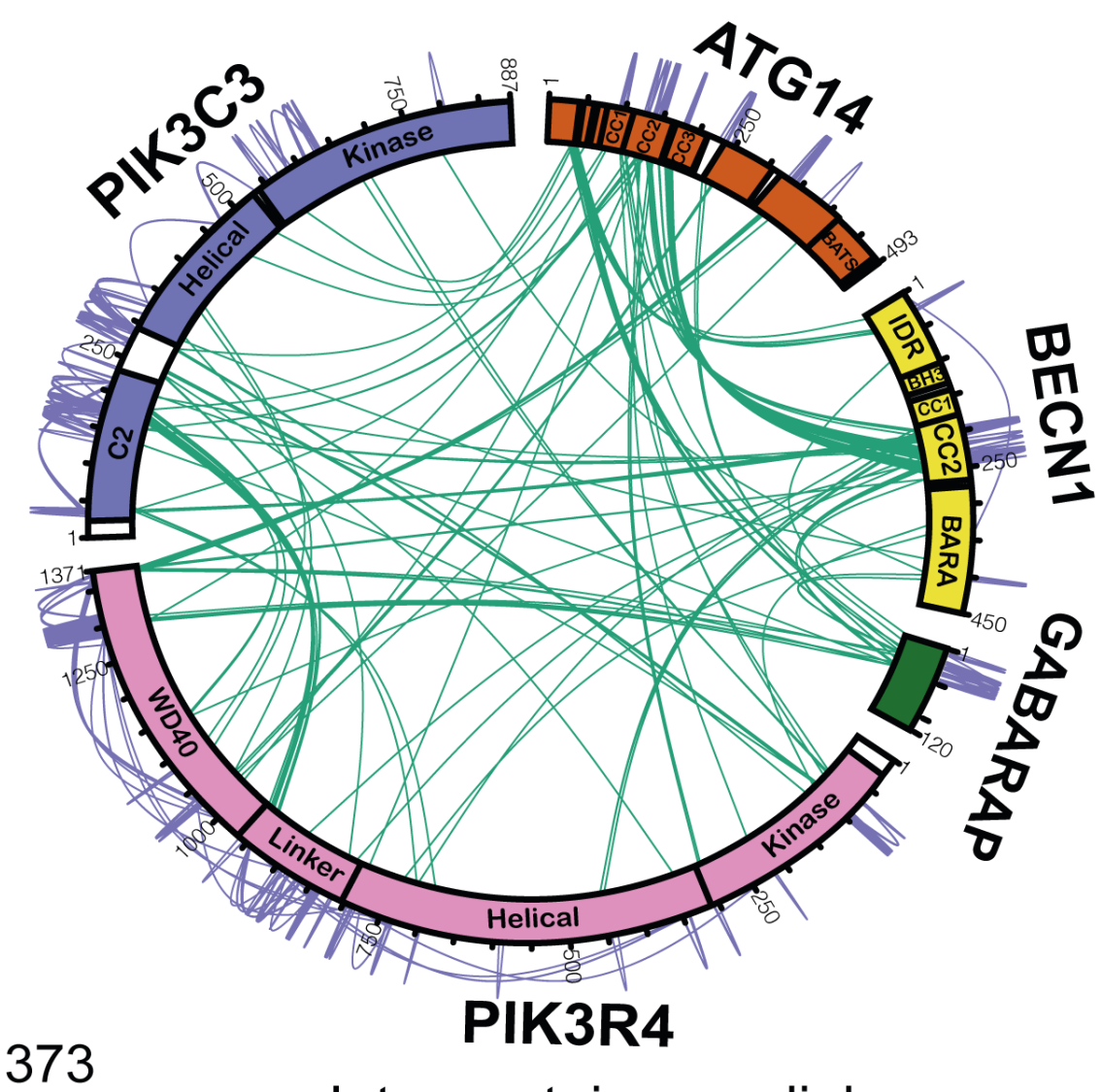

Total intra-protein crosslinks: 373
Total inter-proteins crosslinks: 240
PtdIns3K-C1–GABARAP crosslinks: 32

Intra-protein crosslink
Inter-protein crosslink

Supplementary Figure 3. PtdIns3K-C1 binds GABARAP in the C site and the N site on membranes. Inter-crosslinked peptides between PtdIns3K-C1 and lipidated GABARAP have been identified in both the N site (to ATG14) and the C site (to PIK3R4 and BECN1) in the presence of 100 nm Large Unilamellar Vesicles (LUVs). GABARAP was covalently coupled to the LUVs by maleimide coupling (Cys = cysteine). LUVs inner and outer membrane leaflets are labelled as 'in' and 'out', respectively. All identified crosslinked peptides between PtdIns3K-C1 and GABARAP are mapped on PtdIns3K-C1–GABARAP structure (PDB 9TW2) and on the different protein domains. Numbers of intra-protein crosslinks (blue) and inter-protein crosslinks (green) are indicated.

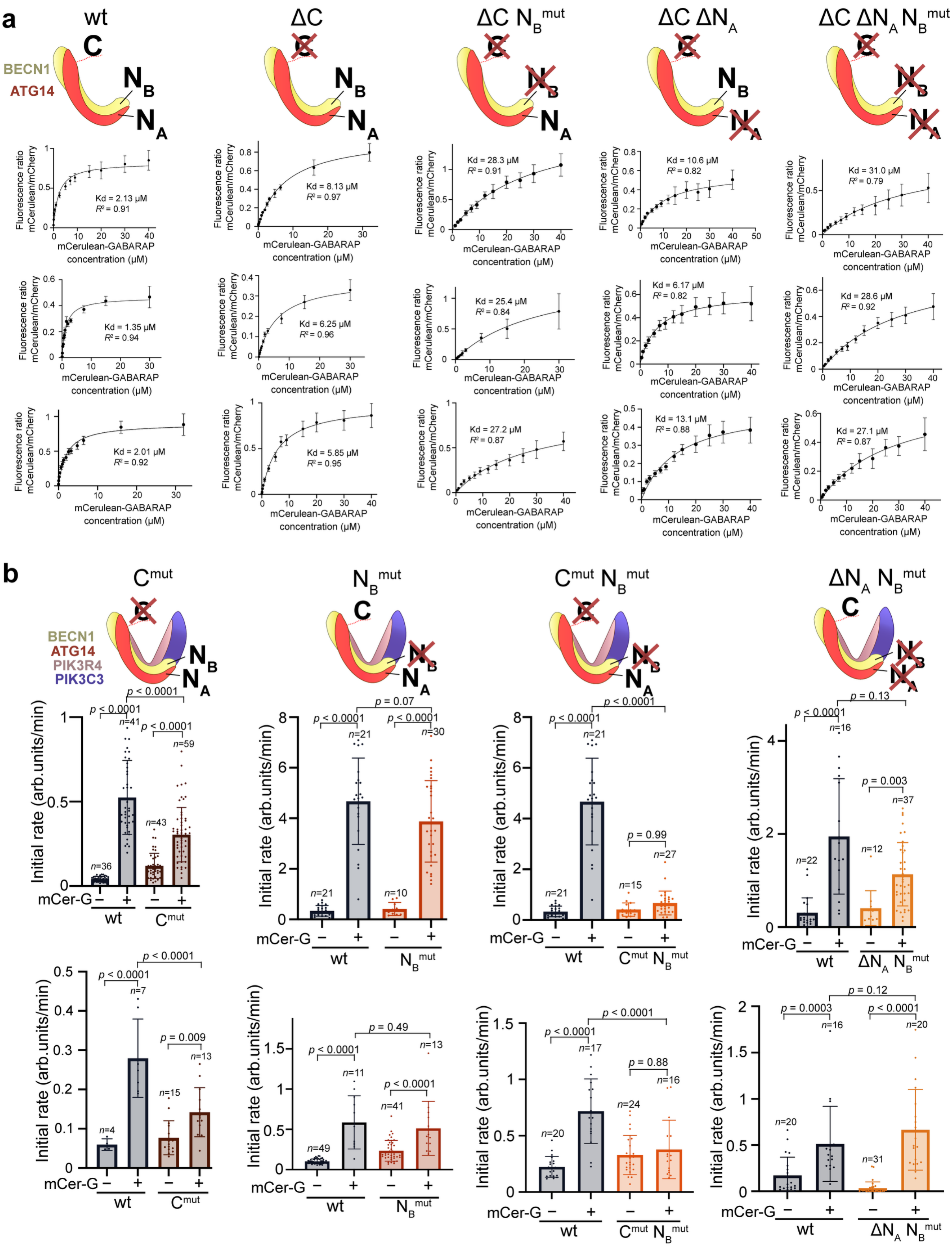

Supplementary Figure 4. The C and N sites cooperate for PtdIns3K-C1 activation by GABARAP. a Replicates of affinity for GABARAP of different C site and N site mutants as shown in Fig. 6b. mCherry-ATG14–BECN1 constructs were immobilized on anti-RFP beads and incubated with different concentrations of mCerulean-GABARAP. Kd and R^2 were determined by a non-linear regression fit. **b** Replicates of GUV-based activity assay of PtdIns3K-C1 carrying mutations on C and N sites in presence or absence of maleimide-lipidated mCerulean-GABARAP (mCer-G), two additional replicates of the experiments shown in Fig. 6c. PtdIns3P production initial rates expressed in AF647-PX fluorescence/min, average ± *SD* for *n* GUVs. Means were compared in a two-way ANOVA with Tukey's correction for multitesting.

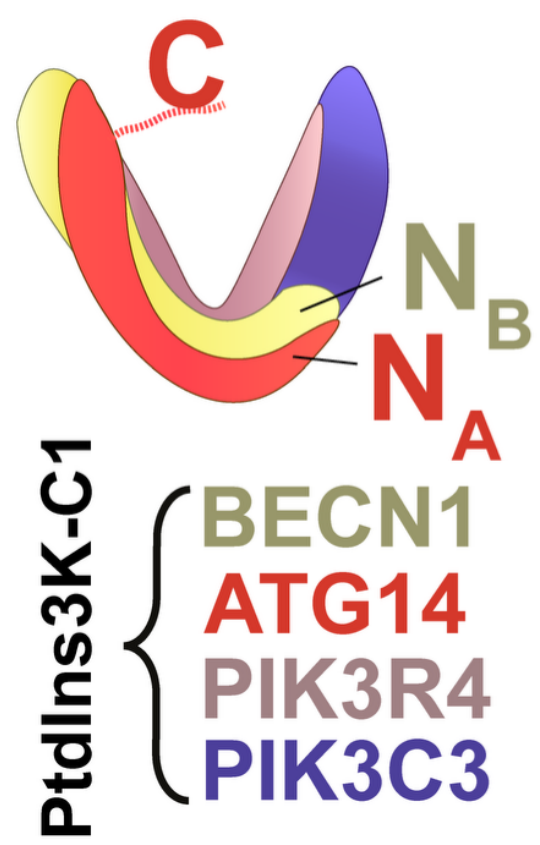

	Site / Experiment	C	N site: N_A	N site: N_B
GABARAP binding sites identification	Cryo-EM (+/– = presence / absence of density)	–	+	+
	HDX-MS (+/– = presence / absence of protection)	+	+	+/– Not covered, protection in neighbouring regions
	XL-MS (+/– = presence / absence of crosslinks)	+	+	–
Functional validation (mutagenesis)	Binding assay (+ = contributes to the binding to GABARAP)	+	+	+
	GUV assay	Contributes to activation	No effect seen on their own, contribute to activation when C site is mutated	

Supplementary Figure 5. Identification and functional validation of several GABARAP-binding sites. Summary of the combination of structural and biochemical approaches allowing the identification of the C site (in ATG14), the N_A site (in ATG14) and the N_B site (in BECN1) of PtdIns3K-C1, and their respective contribution to the activation of PtdIns3K-C1 by lipidated GABARAP.

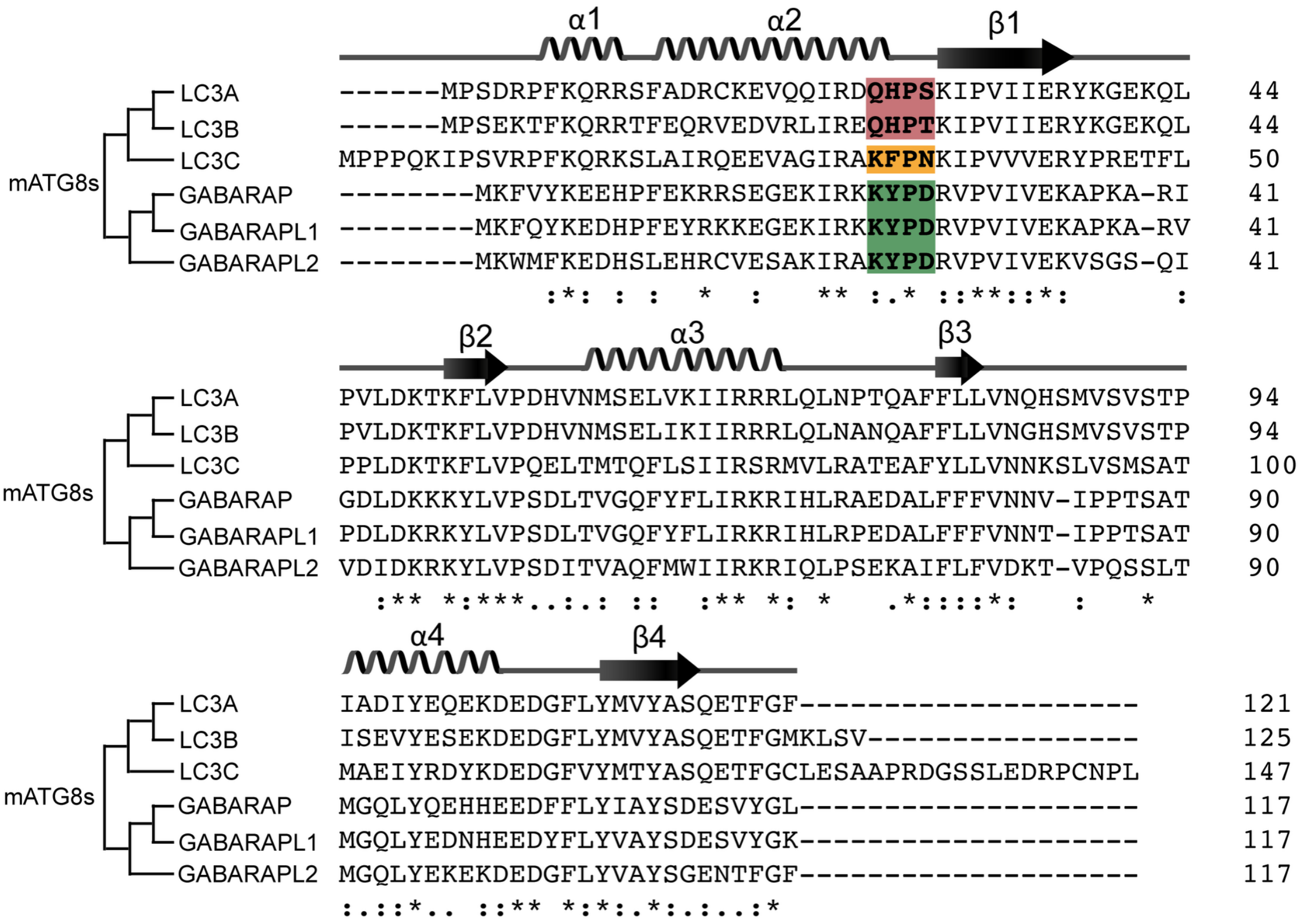

Supplementary Figure 6. LC3 and GABARAP families differ in the α2-β1 loop binding PtdIns3K-C1 N_A site. Multiple sequence alignment using Clustal Omega is overlaid with the secondary structures of GABARAP (PDB 1GNU) and the phylogenic tree of all mATG8s. The KYPD sequence found in GABARAP family is highlighted in green, the similar sequence KFPN of LC3C highlighted in orange, and the QHPS/T sequence of LC3A/B highlighted in red. ‘*’: fully conserved residues, ‘:’: conservation with strongly similar properties, ‘.’: conservation with weakly similar properties, ‘ ‘: non-conserved residues.

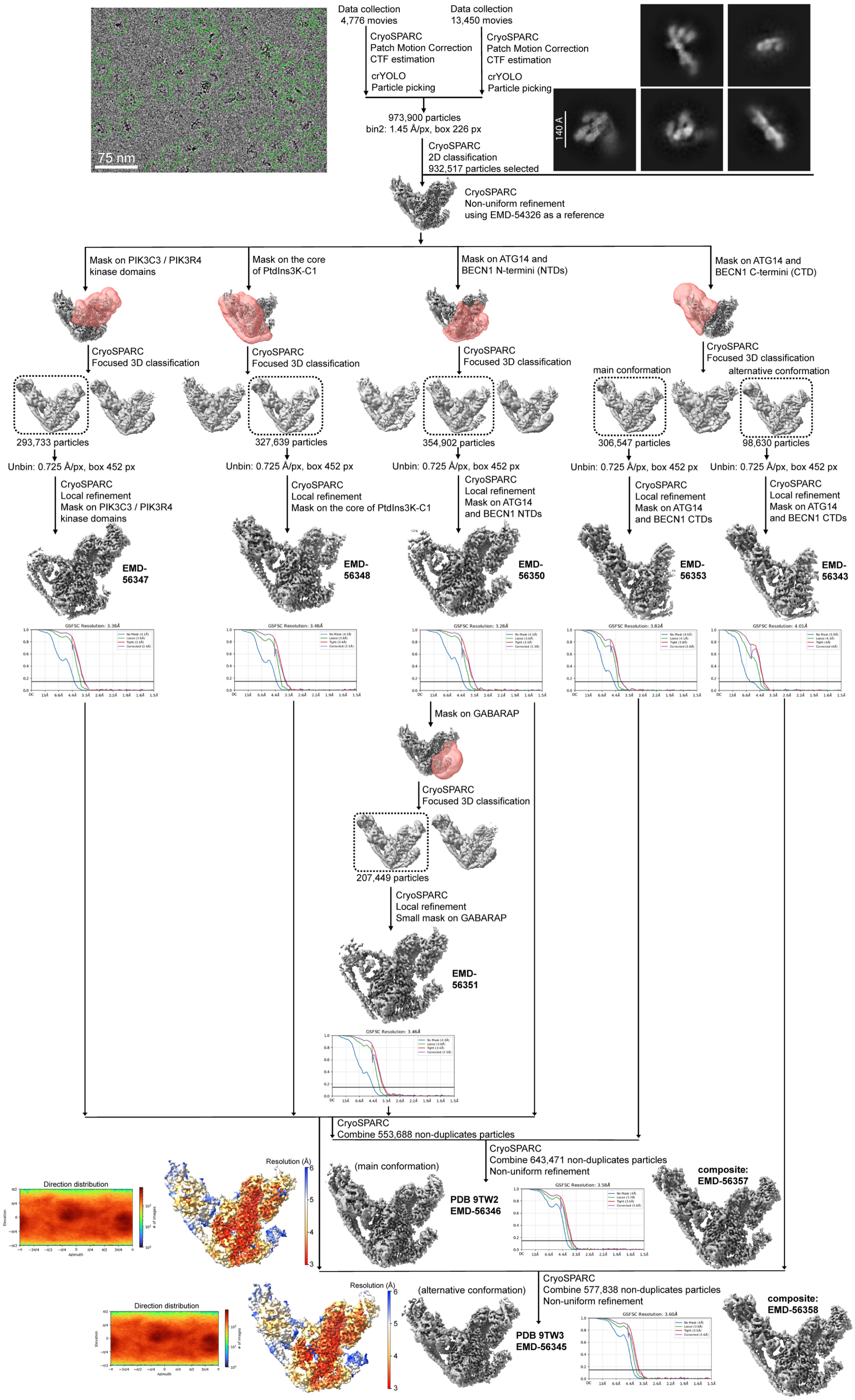

Supplementary Figure 7. Single particle cryo-EM processing workflow for the structure of PtdIns3K-C1 with NRBF2 MIT and GABARAP. CrYOLO picks are circled in green (327 Å diameter) on a representative micrograph. Only a few representative 2D and 3D classes are shown. EMDB accession codes are written under their corresponding maps, as well as the PDB code of the fitted model.

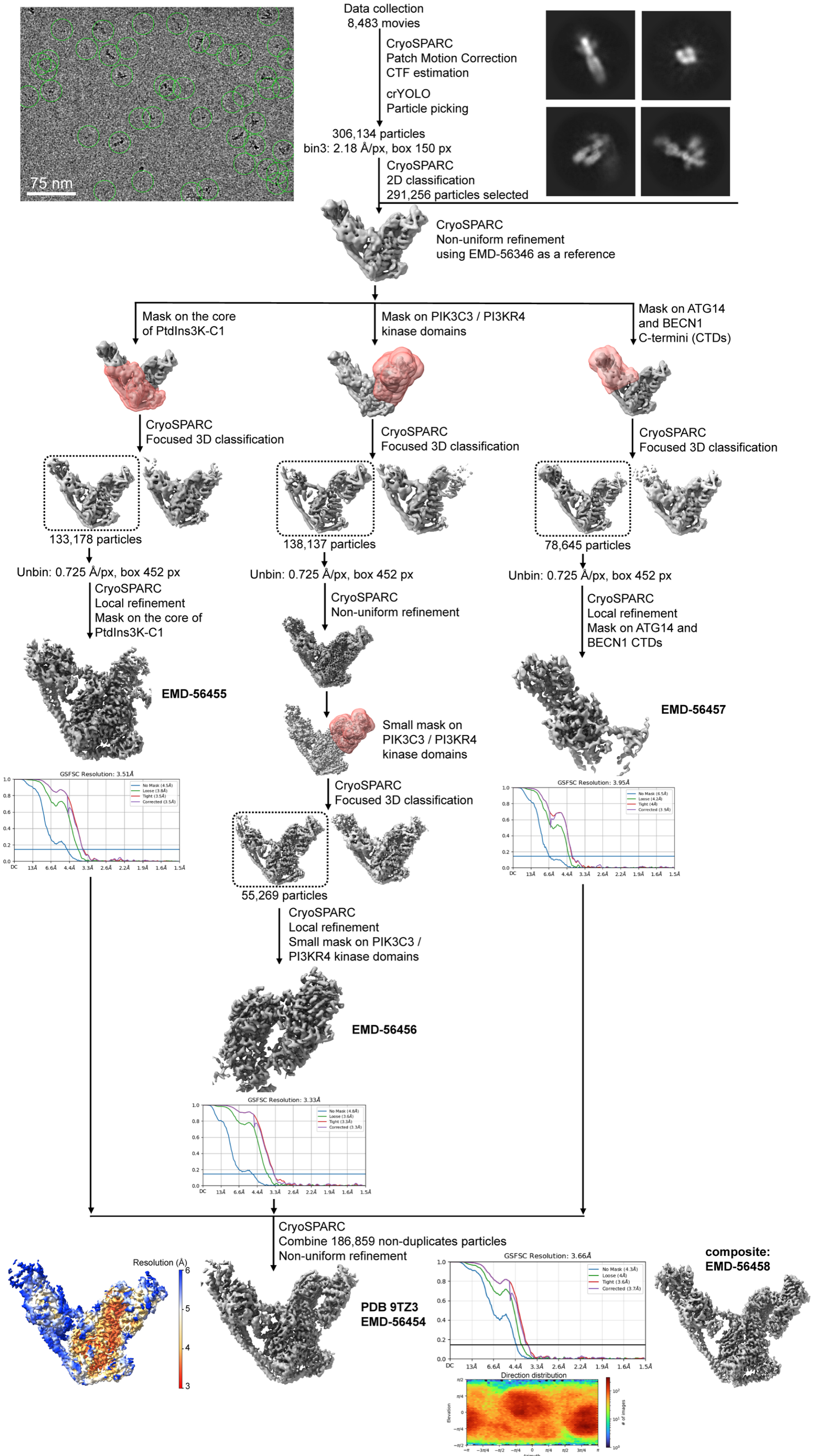

Supplementary Figure 8. Single particle cryo-EM processing workflow for the structure of PtdIns3K-C1 with NRBF2 MIT and ADP-MgF$_3$. CrYOLO picks are circled in green (327 Å diameter) on a representative micrograph. Only a few representative 2D and 3D classes are shown. EMDB accession codes are written under their corresponding maps, as well as the PDB code of the fitted model.
