# Supplementary material for: A GABARAP−PtdIns3K-C1 positive feedback loop at the heart of the phagophore nucleation
Source: Nat Commun. 2026 Jul 30;17:9473. doi: 10.1038/s41467-026-76135-w (PMC13542195; doi:10.1038/s41467-026-76135-w)
Supplement: Supplementary file 2 [file 41467_2026_76135_MOESM2_ESM.pdf]

Description of Additional Supplementary Files

File Name: Supplementary Data 1

Description: HDX-MS information

File Name: Supplementary Data 2

Description: XL-MS information
