# Supplementary material for: A GABARAP−PtdIns3K-C1 positive feedback loop at the heart of the phagophore nucleation
Source: Nat Commun. 2026 Jul 30;17:9473. doi: 10.1038/s41467-026-76135-w (PMC13542195; doi:10.1038/s41467-026-76135-w)
Supplement: Supplementary file 3 [file 41467_2026_76135_MOESM5_ESM.pdf]

nature portfolio

Corresponding author(s): Roger L. Williams

Last updated by author(s): 2/07/2026

Reporting Summary

Nature Portfolio wishes to improve the reproducibility of the work that we publish. This form provides structure for consistency and transparency in reporting. For further information on Nature Portfolio policies, see our Editorial Policies and the Editorial Policy Checklist.

Statistics

For all statistical analyses, confirm that the following items are present in the figure legend, table legend, main text, or Methods section.

n/a	Confirmed	
☐	☒	The exact sample size (*n*) for each experimental group/condition, given as a discrete number and unit of measurement
☐	☒	A statement on whether measurements were taken from distinct samples or whether the same sample was measured repeatedly
☐	☒	The statistical test(s) used AND whether they are one- or two-sided<br>*Only common tests should be described solely by name; describe more complex techniques in the Methods section.*
☒	☐	A description of all covariates tested
☐	☒	A description of any assumptions or corrections, such as tests of normality and adjustment for multiple comparisons
☐	☒	A full description of the statistical parameters including central tendency (e.g. means) or other basic estimates (e.g. regression coefficient) AND variation (e.g. standard deviation) or associated estimates of uncertainty (e.g. confidence intervals)
☐	☒	For null hypothesis testing, the test statistic (e.g. *F*, *t*, *r*) with confidence intervals, effect sizes, degrees of freedom and *P* value noted<br>*Give P values as exact values whenever suitable.*
☒	☐	For Bayesian analysis, information on the choice of priors and Markov chain Monte Carlo settings
☒	☐	For hierarchical and complex designs, identification of the appropriate level for tests and full reporting of outcomes
☒	☐	Estimates of effect sizes (e.g. Cohen's *d*, Pearson's *r*), indicating how they were calculated

Our web collection on statistics for biologists contains articles on many of the points above.

Software and code

Policy information about availability of computer code

Data collection	Fluorescence microscopy experiments were collected on ZEN software (2.1 SP3, Zeiss). Cryo-EM data was collected using EPU (Thermo Fisher). HDX-MS data was collected using a Synapt G2Si-HDMS (Waters), and XL-MS data using an Orbitrap Eclipse (ThermoScientific).
Data analysis	Fluorescence microscopy results were analysed with FIJI software (ImageJ2 2.14.0) and Prism10 (GraphPad) for statistics. Cryo-EM datasets were analysed with cryoSPARC (v4.4), crYOLO-1.7.5, TEMPy-ReFF 1.1, PHENIX 1.21 and UCSF ChimeraX 1.7.1. HDX-MS data was analysed in ProteinLynx Global Server 3.0.1 (PLGS, Waters), HDExaminer 3.4.0 (Trajan Scientific and Medical), Archaeopteryx (https://github.com/Glenn-Masson/Archaeopteryx) and Microsoft Excel (16.109). For XL-MS, ProteoWizard73, XiSearch, and PyMOL 2.5.4 (Schrödinger) used to analyse data.

For manuscripts utilizing custom algorithms or software that are central to the research but not yet described in published literature, software must be made available to editors and reviewers. We strongly encourage code deposition in a community repository (e.g. GitHub). See the Nature Portfolio guidelines for submitting code & software for further information.

Data

Policy information about availability of data

All manuscripts must include a data availability statement. This statement should provide the following information, where applicable:

- Accession codes, unique identifiers, or web links for publicly available datasets
- A description of any restrictions on data availability
- For clinical datasets or third party data, please ensure that the statement adheres to our policy

Plasmids used in this study (Table 1) were deposited on Addgene (https://www.addgene.org/). All the cryo-EM maps have been deposited on the Electron Microscopy Data Bank (EMDB). The cryo-EM data for the GABARAP dataset generated in this study have been deposited in the EMDB database under accession codes: EMD-56357 [https://www.ebi.ac.uk/pdbe/entry/emdb/EMD-56357] (composite map), EMD-56346 [https://www.ebi.ac.uk/pdbe/entry/emdb/EMD-56346] (consensus map), EMD-56347 [https://www.ebi.ac.uk/pdbe/entry/emdb/EMD-56347], EMD-56348 [https://www.ebi.ac.uk/pdbe/entry/emdb/EMD-56348], EMD-56350 [https://www.ebi.ac.uk/pdbe/entry/emdb/EMD-56350], EMD-56351 [https://www.ebi.ac.uk/pdbe/entry/emdb/EMD-56351], EMD-56353 [https://www.ebi.ac.uk/pdbe/entry/emdb/EMD-56353] for the main conformation; and EMD-56358 [https://www.ebi.ac.uk/pdbe/entry/emdb/EMD-56358] (composite map), EMD-56345 [https://www.ebi.ac.uk/pdbe/entry/emdb/EMD-56345] (consensus map) and EMD-56343 [https://www.ebi.ac.uk/pdbe/entry/emdb/EMD-56343] for the adaptor arm alternative conformation (see Supplementary Fig. 7). The two cryo-EM atomic models generated in this study have been deposited in the Protein Data Bank (PDB) database under accession codes 9TW2 [https://doi.org/10.2210/pdb9TW2/pdb] (main conformation) and 9TW3 [https://doi.org/10.2210/pdb9TW3/pdb]) (alternative conformation). The cryo-EM data of the ADP-containing dataset generated in this study have been deposited in the EMDB database under accession codes EMD-56458 [https://www.ebi.ac.uk/pdbe/entry/emdb/EMD-56458] (composite map), EMD-56454 [https://www.ebi.ac.uk/pdbe/entry/emdb/EMD-56454] (consensus map), EMD-56455 [https://www.ebi.ac.uk/pdbe/entry/emdb/EMD-56455], EMD-56456 [https://www.ebi.ac.uk/pdbe/entry/emdb/EMD-56456], EMD-56457 [https://www.ebi.ac.uk/pdbe/entry/emdb/EMD-56457] (see Supplementary Fig. 8). The atomic model was deposited in the PDB database under accession code 9TZ3 [https://doi.org/10.2210/pdb9TZ3/pdb]. HDX-MS and XL-MS data generated in this study have been deposited in the ProteomeXchange Consortium via the PRIDE partner repository database under the accession codes PXD074963 [http://proteomecentral.proteomexchange.org/cgi/GetDataset?ID=PXD074963] and PXD079187 [http://proteomecentral.proteomexchange.org/cgi/GetDataset?ID=PXD079187]. The Deuteration tables generated in this study are provided in Supplementary Data 1, and crosslinks tables in Supplementary Data 2. Source Data are provided with this paper.

Research involving human participants, their data, or biological material

Policy information about studies with human participants or human data. See also policy information about sex, gender (identity/presentation), and sexual orientation and race, ethnicity and racism.

Reporting on sex and gender	*Use the terms sex (biological attribute) and gender (shaped by social and cultural circumstances) carefully in order to avoid confusing both terms. Indicate if findings apply to only one sex or gender; describe whether sex and gender were considered in study design; whether sex and/or gender was determined based on self-reporting or assigned and methods used.*<br>*Provide in the source data disaggregated sex and gender data, where this information has been collected, and if consent has been obtained for sharing of individual-level data; provide overall numbers in this Reporting Summary. Please state if this information has not been collected.*<br>*Report sex- and gender-based analyses where performed, justify reasons for lack of sex- and gender-based analysis.*
Reporting on race, ethnicity, or other socially relevant groupings	*Please specify the socially constructed or socially relevant categorization variable(s) used in your manuscript and explain why they were used. Please note that such variables should not be used as proxies for other socially constructed/relevant variables (for example, race or ethnicity should not be used as a proxy for socioeconomic status).*<br>*Provide clear definitions of the relevant terms used, how they were provided (by the participants/respondents, the researchers, or third parties), and the method(s) used to classify people into the different categories (e.g. self-report, census or administrative data, social media data, etc.)*<br>*Please provide details about how you controlled for confounding variables in your analyses.*
Population characteristics	*Describe the covariate-relevant population characteristics of the human research participants (e.g. age, genotypic information, past and current diagnosis and treatment categories). If you filled out the behavioural & social sciences study design questions and have nothing to add here, write "See above."*
Recruitment	*Describe how participants were recruited. Outline any potential self-selection bias or other biases that may be present and how these are likely to impact results.*
Ethics oversight	*Identify the organization(s) that approved the study protocol.*

Note that full information on the approval of the study protocol must also be provided in the manuscript.

Field-specific reporting

Please select the one below that is the best fit for your research. If you are not sure, read the appropriate sections before making your selection.

[x] Life sciences [ ] Behavioural & social sciences [ ] Ecological, evolutionary & environmental sciences

For a reference copy of the document with all sections, see nature.com/documents/nr-reporting-summary-flat.pdf

Life sciences study design

All studies must disclose on these points even when the disclosure is negative.

Sample size	Samples sizes were chosen with the highest sample size possible. For cryo-EM, the sample size is sufficient to obtain a structure at the reported resolution, as assessed by Fourier shell correlation. For HDX and kinetic assays, we made sufficient independent measurements to estimate the error of the measurements. For GUV assays, the sample size corresponds to the number of vesicles that were analyzed and is listed on each figure with GUV results. For bead assays, the sample size corresponds to the number of beads that were analyzed and is described on each figure with results.
Data exclusions	For HDX, peptide inclusion criteria were a minimum fragment per amino acid of 0.2, a minimum intensity of 103, a peptide length between 5 and 25 residues, a minimum PLGS score of 6.62, and an MH+ tolerance of 5 ppm. For XL-MS, search conditions used 3 maximum missed cleavages with a minimum peptide length of 5. Variable modifications used were carbmidomethylation of cysteine (57.02146 Da) and Methionine oxidation (15.99491 Da). False discovery rate was set to 5%.
Replication	Assays were done in three technical replicates. For cryo-EM, structures were determined from independent half datasets, which were compared to assess the resolution of the reconstruction, as described in the methods section.
Randomization	For Cryo-EM, division of dataset into two random halves was done based on particle number.
Blinding	Blinding was not applicable to this study because this type of study does not use group allocation.

Reporting for specific materials, systems and methods

We require information from authors about some types of materials, experimental systems and methods used in many studies. Here, indicate whether each material, system or method listed is relevant to your study. If you are not sure if a list item applies to your research, read the appropriate section before selecting a response.

Materials & experimental systems

n/a	Involved in the study	
☐	☒	Antibodies
☐	☒	Eukaryotic cell lines
☒	☐	Palaeontology and archaeology
☒	☐	Animals and other organisms
☒	☐	Clinical data
☒	☐	Dual use research of concern
☒	☐	Plants

Methods

n/a	Involved in the study	
☒	☐	ChIP-seq
☒	☐	Flow cytometry
☒	☐	MRI-based neuroimaging

Antibodies

Antibodies used	Antibodies against endogenous LC3 (Caltag Medsystems MBL-PM036), FIP200 (ProteinTech 10069-1-AP) or WIPI2 (Bio Rad LaboratoriesMCA5780GA), all at 1:100 dilution.
Validation	Tabata, K., et al., Mol. Biol. Cell 21, 4162-4172 (2010). Lee, D., & Goldberg, A. L. (2022). Proceedings of the National Academy of Sciences, 119(25), e2122482119. Polson, H.E. et al. (2010) Autophagy. 6 (4): 506-22.

Eukaryotic cell lines

Policy information about cell lines and Sex and Gender in Research

Cell line source(s)	HEK293 (ATCC CRL-1573). Expi293 suspension cells (ThermoFisher A14527), S. cerevisiae YOY193 (Matα pep4::HIS3 prb::LEU2 bar1:HISG lys2::GAL1/10-GAL4 can1 ade2 ura3 leu2–3,112 trp1 TRX1-ADE2::ade2Δ) (Rostislavleva et al., 2015).
Authentication	All cell lines were used without authentication
Mycoplasma contamination	The cell lines for protein production were not routinely tested for mycoplasma contamination.
Commonly misidentified lines (See ICLAC register)	None

Plants

Seed stocks	*Report on the source of all seed stocks or other plant material used. If applicable, state the seed stock centre and catalogue number. If plant specimens were collected from the field, describe the collection location, date and sampling procedures.*
Novel plant genotypes	*Describe the methods by which all novel plant genotypes were produced. This includes those generated by transgenic approaches, gene editing, chemical/radiation-based mutagenesis and hybridization. For transgenic lines, describe the transformation method, the number of independent lines analyzed and the generation upon which experiments were performed. For gene-edited lines, describe the editor used, the endogenous sequence targeted for editing, the targeting guide RNA sequence (if applicable) and how the editor was applied.*
Authentication	*Describe any authentication procedures for each seed stock used or novel genotype generated. Describe any experiments used to assess the effect of a mutation and, where applicable, how potential secondary effects (e.g. second site T-DNA insertions, mosiacism, off-target gene editing) were examined.*
