# Supplementary material for: A GABARAP−PtdIns3K-C1 positive feedback loop at the heart of the phagophore nucleation
Source: Nat Commun. 2026 Jul 30;17:9473. doi: 10.1038/s41467-026-76135-w (PMC13542195; doi:10.1038/s41467-026-76135-w)
Supplement: Supplementary file 4 [file 41467_2026_76135_MOESM6_ESM.pdf]

nature portfolio

Peer Review File

A GABARAP−PtdIns3K-C1 positive feedback loop at the heart of the phagophore nucleation

Corresponding Author: Dr Roger Williams

This file contains all reviewer reports in order by version, followed by all author rebuttals in order by version.

Version 0:

Reviewer comments:

Reviewer #1

(Remarks to the Author)
The present manuscript by Dessus et al entitled "A GABARAP−PtdIns3K-C1 positive feedback loop at the heart of the phagophore nucleation" presents data demonstrating a role for GABARAP in setting up a positive feedback loop to enable the generation of enough membrane to form many large autophagosomes within a short time frame upon induction of macroautophagy by binding to two sites in the PtdIns3K-C1 complex. The authors assembled the PtdIns3K-C1 complex consisting of the PIK3C3 lipid kinase (VPS34), the PIK3R4 pseudokinase (VPS15), ATG14 (BAKOR) and BECN1 in vitro and used a combination of cryo-electron microscopy, structural mass spectrometry (HDX-MS and XL-MS), activity assays on GUVs (giant unilamellar vesicles) and mutagenesis to show that GABARAP binds two sites in PtdIns3K-C1 and activates the kinase to produce PdIns3P and sets up a positive feedback loop.

This is an elegant, very interesting and clearly very important study with convincing data throwing light on the role of ATG8 family proteins, or more specifically GABARAP in particular, in interacting with the PtdIns3K-C1 complex of the autophagy core machinery to amplify phagophore nucleation and expansion. The authors confirm that the previously characterized LIRs in ATG14L and BECN1 (Ref. 23) are important in the assembled PtdIns3K-C1 complex and very significantly they show that a positive feedback loop is formed where PtdIns3K-C1 interacts with GABARAP and becomes activated to produce more PtdIns3P, thereby further stimulating GABARAP lipidation. This is important for explaining the rapid formation of many autophagosomes upon induction of macroautophagy by mTOR inhibition for example upon nutrient starvation. Another interesting aspect of the study is that the authors found the BECN1 LIR (residues 97-100) to be part of a bipartite GABARAP binding site where the other part of the binding site is provided by the N-terminal residues 37-40 of ATG14L. Such a bipartite ATG8 family binding site where two different proteins contribute has not been described before.

A few minor comments:
1. In the Introduction the authors write: "Autophagy is initiated at the endoplasmic reticulum (ER) where stress signals are integrated by the unc-51 like kinase complex ULK1, which recruits the class III PI3K complex 1 (hereafter referred to as PtdIns3K-C1)9,10." In a BioRxiv paper from the Hurley lab https://doi.org/10.1101/2025.11.07.687251) there is mentioned that the ULK1C and the PI3KC1 is co-recruited. Would the authors like to comment on this?

2. In the second paragraph of the Result section the authors write that "Titration gave an apparent Kd of 1.2 µM for the interaction between PtdIns3K-C1 and GABARAP (Fig. 2c, Supplementary Fig. 1a)." In the Discussion section it could be commented that this is close to the Kd of 2.8 µM determined for the binding of GABARAP to the ATG14 LIR peptide by Birgisdottir et al. (Ref. 23). Also the Kd measurements in Fig 6B and Suppl. Fig. 4A done with bead-binding assays using ATG14-BECN1 heterodimers WT and with LIR mutations are worth discussing relative to the Kds determined for the isolated peptides of the ATG14L1 LIR and the BECN1 LIR as the values are very close.

3. Both based on binding affinities, and, more importantly, based on activation assays the C LIR (ATG14L1 LIR) was found to be the main contributor to the activation and the resulting positive feedback loop. In their Discussion the authors "propose that this selectivity of PtdIns3K-C1 for the GABARAP family of mATG8s arises from the specific residues at the contact of the NA site we discovered." This is difficult to accept for several reasons. The C LIR is the stronger GABARAP binder and main contributor to activation. The C LIR is binding preferentially to GABARAP too. In ref. 23 Birgisdottir et al. present structural arguments for the variation in hydrophobic pocket 2 to explain why both the BECN1 and ATG14 LIR bind preferentially to GABARAP family proteins. These two views should be mentioned and discussed in the Discussion section.

Reviewer #2

(Remarks to the Author)
In this manuscript, Dessus et al. reveal the cryo-EM structure of PI3K complex I (PI3KC1) together with GABARAP. Initially, they characterized a cellular condition of a defect in the Atg8-lipidation machinery, and characterized the increase in cellular punctae WIPI2 and FIP200. In vitro binding analysis of all six human mCerulean-mATG8 proteins on beads and GUVs, using recombinant ATG7 (E1), ATG3 (E2), the ATG12-ATG5–ATG16L1 (E3) complex, revealed that there are distinct affinities between various Atg8-family proteins such as LC3A/B/C and GABARAP/L1/L2 and the binding of them to PI3KC1. GABARAPL2 was found to be most efficiently binding. While the cryo-EM structure reveals GABARAP bound to N-termini of ATG14 and BECN1 at the N-site, cross-linking and hydrogen-deuterium exchange show alternative binding to the other side of the V-shaped molecule the C-side. Activity assays and mutagenesis support the idea of a positive feedback loop between both GABARAP binding sites stimulating PI3 kinase activity.

The structural findings regarding the two cooperatively acting GABARAP binding sites are novel and important for the field of autophagy and beyond. The cryo-EM structures of PI3KC1 withGABARAP and ADP:MgF3 included in this manuscript are challenging to perform. The work is well presented, the refinement strategy highly suitable including the local refinement maps using masks and the resulting map qualities are very good. The conclusions are clearly supported by the presented data and, therefore, the publication is ready for publication at Nature Communications without much delay. Only minor comments were noted.

Minor:
1. Supplementary Fig. 6/7 present the image processing workflow clearly. The two micrographs at the top are, however, highly noisy and particles almost impossible to discern. Please add a scale bar and encircle detected particles for improved comprehension.
2. In the discussion, the authors attempt to rationalize the observed differences in binding affinity of the mAtg8 family proteins by multiple sequence alignment from a structural point of view. Moreover, it is intriguing to discuss additional possibilities on the observed affinity differences of various mAtg8 family proteins such as differences in cell-specific expression levels etc.
3. Lipdation mutant phenotypes.
The Atg5 K.O. phenotype goes back quite a long way and original work of the Mizoshima group should be cited (DOI: 10.1038/nature03029) and the role of ATG3 K.O. (10.1038/s41586-020-2992-3) described. Although originally tracked back to the lipidiation machinery, it is likely that Atg5,3,7 K.O. have specific phenotypes each. Therefore, a more detailed treatment of these aspects in the introduction is warranted.

Reviewer #3

(Remarks to the Author)
This manuscript describes a positive feedback mechanism between lipidated mammalian ATG8 proteins and PtdIns3K-C1 during phagophore nucleation. The central finding is that membrane-coupled mATG8s, particularly GABARAP and GABARAPL1, bind to and activate PtdIns3K-C1, thereby increasing PtdIns3P production and potentially reinforcing WIPI recruitment and further mATG8 lipidation. The authors combine cell imaging, reconstituted membrane assays, cryo-EM, HDX-MS, XL-MS and mutagenesis to identify two GABARAP-binding sites on PtdIns3K-C1 and to show that these sites cooperate in kinase activation. The work addresses an important conceptual gap in autophagy: how early phagophore-associated signals are amplified rapidly to support autophagosome biogenesis. The study is technically strong, and the overall model is compelling. The in vitro reconstitution is particularly persuasive. The authors show that lipidated, but not soluble, GABARAP strongly activates PtdIns3K-C1, and they connect this activity to defined binding sites using structure-guided mutagenesis.
The structural proteomics is a nice example of integrating these tehcniques. HDX-MS is used appropriately to interrogate regions of PtdIns3K-C1 that are flexible or not resolved by cryo-EM, while photo-XL-MS provides residue-level proximity evidence for both the cryo-EM-defined N site and the flexible C-terminal ATG14 LIR-containing site. The authors then validate these regions by mutagenesis and activity assays.

Comments
1. In the crosslinking mass spectrometry methods, I assume that the peptides were fractionated by size exclusion chromatography in 30% acetonitrile *and* 0.1% TFA. Otherwise you will have retained a lot of peptides by charge.
2. A table of crosslinked residue pairs along with scores etc should be included with the manuscript.
3. It is best practice to upload mzidentML to PRIDE. This can also be output from XiFDR.
4. Crosslinks should be mapped on the Ptdins3K-C1 structure in at least one figure.
5. The cryo-EM and HDX-MS descriptions use several site names: NA, NB, N site and C site. These are logical, but the nomenclature becomes dense. A single schematic showing all sites, whether each is visible by cryo-EM, protected by HDX-MS, crosslinked by XL-MS and functionally tested by mutagenesis might help the reader, but is not necessary.
6. The model would benefit from a clearer comparison with the previously described WIPI2-PtdIns3K-C1 feedback mechanism motioned.
7. The authors should define more explicitly whether the increased WIPI2/FIP200 ratio or puncta intensity is being used as a proxy for PtdIns3P production, PtdIns3P persistence, or recruitment efficiency. These are related but not identical.

Reviewer #4

(Remarks to the Author)
I co-reviewed this manuscript with one of the reviewers who provided the listed reports. This is part of the Nature Communications initiative to facilitate training in peer review and to provide appropriate recognition for Early Career Researchers who co-review manuscripts.

Version 1:

Reviewer comments:

Reviewer #1

(Remarks to the Author)
The authors have addressed my comments to my satisfaction.

Reviewer #2

(Remarks to the Author)
My raised points have been answered satisfactorily.

Reviewer #3

(Remarks to the Author)
The Authors have addressed all my concerns.

Reviewer #4

(Remarks to the Author)
I co-reviewed this manuscript with one of the reviewers who provided the listed reports. This is part of the Nature Communications initiative to facilitate training in peer review and to provide appropriate recognition for Early Career Researchers who co-review manuscripts.

Open Access This Peer Review File is licensed under a Creative Commons Attribution 4.0 International License, which permits use, sharing, adaptation, distribution and reproduction in any medium or format, as long as you give appropriate credit to the original author(s) and the source, provide a link to the Creative Commons license, and indicate if changes were made.
In cases where reviewers are anonymous, credit should be given to 'Anonymous Referee' and the source.
The images or other third party material in this Peer Review File are included in the article’s Creative Commons license, unless indicated otherwise in a credit line to the material. If material is not included in the article’s Creative Commons license and your intended use is not permitted by statutory regulation or exceeds the permitted use, you will need to obtain permission directly from the copyright holder.
To view a copy of this license, visit https://creativecommons.org/licenses/by/4.0/

ANSWERS TO REVIEWERS COMMENTS

Reviewer #1 (Remarks to the Author):

The present manuscript by Dessus et al entitled "A GABARAP−PtdIns3K-C1 positive feedback loop at the heart of the phagophore nucleation" presents data demonstrating a role for GABARAP in setting up a positive feedback loop to enable the generation of enough membrane to form many large autophagosomes within a short time frame upon induction of macroautophagy by binding to two sites in the PtdIns3K-C1 complex. The authors assembled the PtdIns3K-C1 complex consisting of the PIK3C3 lipid kinase (VPS34), the PIK3R4 pseudokinase (VPS15), ATG14 (BAKOR) and BECN1 in vitro and used a combination of cryo-electron microscopy, structural mass spectrometry (HDX-MS and XL-MS), activity assays on GUVs (giant unilamellar vesicles) and mutagenesis to show that GABARAP binds two sites in PtdIns3K-C1 and activates the kinase to produce PdIns3P and sets up a positive feedback loop.

This is an elegant, very interesting and clearly very important study with convincing data throwing light on the role of ATG8 family proteins, or more specifically GABARAP in particular, in interacting with the PtdIns3K-C1 complex of the autophagy core machinery to amplify phagophore nucleation and expansion. The authors confirm that the previously characterized LIRs in ATG14L and BECN1 (Ref. 23) are important in the assembled PtdIns3K-C1 complex and very significantly they show that a positive feedback loop is formed where PtdIns3K-C1 interacts with GABARAP and becomes activated to produce more PtdIns3P, thereby further stimulating GABARAP lipidation. This is important for explaining the rapid formation of many autophagosomes upon induction of macroautophagy by mTOR inhibition for example upon nutrient starvation. Another interesting aspect of the study is that the authors found the BECN1 LIR (residues 97-100) to be part of a bipartite GABARAP binding site where the other part of the binding site is provided by the N-terminal residues 37-40 of ATG14L. Such a bipartite ATG8 family binding site where two different proteins contribute has not been described before.

A few minor comments:

1. In the Introduction the authors write: "Autophagy is initiated at the endoplasmic reticulum (ER) where stress signals are integrated by the unc-51 like kinase complex ULK1, which recruits the class III PI3K complex 1 (hereafter referred to as PtdIns3K-C1)9,10." In a BioRxiv paper from the Hurley lab https://doi.org/10.1101/2025.11.07.687251) there is mentioned that the ULK1C and the PI3KC1 is co-recruited. Would the authors like to comment on this?

We appreciate that the reviewer pointed out this preprint, and we have now cited this in the Discussion among the other proposed positive feedback loops (page 5 line 61). While this preprint mentions co-recruitment of ULK1 and VPS34-C1, co-recruitment was not demonstrated there but in a previous study (PMID 40442316). We have revised the introduction (page 2, line 7) so that it now mentions the co-recruitment of ULK1 and PtdIns3K-C1. This preprint paper of Duan et al. showed that the PI(3)P-binding WIPI proteins recruit the ATG13-ATG101 heterodimer, which is part of the ULK1 complex, to the membrane surface. Similarly to their WIPI2 paper (PMID: 32437499), which we described in the introduction, the authors detected protein-protein interactions but did not measure the PtdIns3K-C1 activity when they bound. To our knowledge, our present paper is the first to measure the PtdIns3K-C1 activity when it associated with other Atg proteins.

2. In the second paragraph of the Result section the authors write that "Titration gave an apparent Kd of 1.2 µM for the interaction between PtdIns3K-C1 and GABARAP (Fig. 2c, Supplementary Fig. 1a)." In the Discussion section it could be commented that this is close to the Kd of 2.8 µM determined for the binding of GABARAP to the ATG14 LIR peptide by Birgisdottir et al. (Ref. 23). Also the Kd measurements in Fig 6B and Suppl. Fig. 4A done with bead-binding assays using ATG14-BECN1 heterodimers WT and with LIR mutations are worth discussing relative to the Kds determined for the isolated peptides of the ATG14L1 LIR and the BECN1 LIR as the values are very close.

A comparison with the Kd values obtained in Birgisdottir et al. was added to the Discussion (page 5 line 7).

3. Both based on binding affinities, and, more importantly, based on activation assays the C LIR (ATG14L1 LIR) was found to be the main contributor to the activation and the resulting positive feedback loop. In their Discussion the authors "propose that this selectivity of PtdIns3K-C1 for the GABARAP family of mATG8s arises from the specific residues at the contact of the NA site we discovered." This is difficult to accept for several reasons. The C LIR is the stronger GABARAP binder and main contributor to activation. The C LIR is binding preferentially to GABARAP too. In ref. 23 Birgisdottir et al. present structural arguments for the variation in hydrophobic pocket 2 to explain why both the BECN1 and ATG14 LIR bind preferentially to GABARAP family proteins. These two views should be mentioned and discussed in the Discussion section.

The reviewer has made an excellent point. In our discussion we had focused on the GABARAP-specific NA interactions apparent from our structure of GABARAP with the full PtdIns3K-C1. However, the reviewer is correct that previously characterised peptide/mATG8 interactions (C site and NB site), are structurally suited to preferential GABARAP binding. We have updated our discussion so that both hypotheses are now discussed side by side (page 5 line 33).

Reviewer #2 (Remarks to the Author):

In this manuscript, Dessus et al. reveal the cryo-EM structure of PI3K complex I (PI3KC1) together with GABARAP. Initially, they characterized a cellular condition of a defect in the Atg8-lipidation machinery, and characterized the increase in cellular punctae WIPI2 and FIP200. In vitro binding analysis of all six human mCerulean-mATG8 proteins on beads and GUVs, using recombinant ATG7 (E1), ATG3 (E2), the ATG12-ATG5–ATG16L1 (E3) complex, revealed that there are distinct affinities between various Atg8-family proteins such as LC3A/B/C and GABARAP/L1/L2 and the binding of them to PI3KC1. GABARAPL2 was found to be most efficiently binding. While the cryo-EM structure reveals GABARAP bound to N-termini of ATG14 and BECN1 at the N-site, cross-linking and hydrogen-deuterium exchange show alternative binding to the other side of the V-shaped molecule the C-side. Activity assays and mutagenesis support the idea of a

positive feedback loop between both GABARAP binding sites stimulating PI3 kinase activity.

The structural findings regarding the two cooperatively acting GABARAP binding sites are novel and important for the field of autophagy and beyond. The cryo-EM structures of PI3KC1 withGABARAP and ADP:MgF3 included in this manuscript are challenging to perform. The work is well presented, the refinement strategy highly suitable including the local refinement maps using masks and the resulting map qualities are very good. The conclusions are clearly supported by the presented data and, therefore, the publication is ready for publication at Nature Communications without much delay. Only minor comments were noted.

Minor:
1. Supplementary Fig. 6/7 present the image processing workflow clearly. The two micrographs at the top are, however, highly noisy and particles almost impossible to discern. Please add a scale bar and encircle detected particles for improved comprehension.

We thank the reviewer for spotting this. We had mistakenly included two binned images. We have now replaced them with two full-scale images with the particles encircled. These are now shown in Supplementary Fig. 7 and 8.

2. In the discussion, the authors attempt to rationalize the observed differences in binding affinity of the mAtg8 family proteins by multiple sequence alignment from a structural point of view. Moreover, it is intriguing to discuss additional possibilities on the observed affinity differences of various mAtg8 family proteins such as differences in cell-specific expression levels etc.

This is an interesting point. A recent study on multiplex genome tagging of GABARAP proteins revealed they localised to different membrane compartments and associated with different proteins (PMID: 39433127). This means that the local concentrations of GABARAPs are different among the membrane compartments, which could result in different levels of association with PtdIns3K-C1. These parameters will fine-tune the PtdIns3K-C1-GABRARAPs feedback mechanism in the cell. However, given that we have no direct observations concerning this fine-tuning, we do not feel further discussion is warranted.

3. Lipdation mutant phenotypes.
The Atg5 K.O. phenotype goes back quite a long way and original work of the Mizoshima group should be cited (DOI: 10.1038/nature03029) and the role of ATG3 K.O. (10.1038/s41586-020-2992-3) described. Although originally tracked back to the lipidiation machinery, it is likely that Atg5,3,7 K.O. have specific phenotypes each. Therefore, a more detailed treatment of these aspects in the introduction is warranted.

In this manuscript we did not expand on the specific cellular roles of ATG3, ATG5, ATG7 and their phenotypes as KO in mice, as these proteins only play a minor role in our manuscript contrarily with lipidated mATG8s and their feedback mechanism with PtdIns3K-C1. We agree with the reviewer that these questions are important in autophagy, as other aspects like the scaffold these proteins were suggested to form on membranes (PMID: 24485455), but as we do not show any characterisation of these enzymes in our manuscript, we think they would rather be distracting if too detailed in the introduction.

Reviewer #3 (Remarks to the Author):

This manuscript describes a positive feedback mechanism between lipidated mammalian ATG8 proteins and PtdIns3K-C1 during phagophore nucleation. The central finding is that membrane-coupled mATG8s, particularly GABARAP and GABARAPL1, bind to and activate PtdIns3K-C1, thereby increasing PtdIns3P production and potentially reinforcing WIPI recruitment and further mATG8 lipidation. The authors combine cell imaging, reconstituted membrane assays, cryo-EM, HDX-MS, XL-MS and mutagenesis to identify two GABARAP-binding sites on PtdIns3K-C1 and to show that these sites cooperate in kinase activation. The work addresses an important conceptual gap in autophagy: how early phagophore-associated signals are amplified rapidly to support autophagosome biogenesis. The study is technically strong, and the overall model is compelling. The in vitro reconstitution is particularly persuasive. The authors show that lipidated, but not soluble, GABARAP strongly activates PtdIns3K-C1, and they connect this activity to defined binding sites using structure-guided mutagenesis.
The structural proteomics is a nice example of integrating these tehcniques. HDX-MS is used appropriately to interrogate regions of PtdIns3K-C1 that are flexible or not resolved by cryo-EM, while photo-XL-MS provides residue-level proximity evidence for both the cryo-EM-defined N site and the flexible C-terminal ATG14 LIR-containing site. The authors then validate these regions by mutagenesis and activity assays.

Comments
1. In the crosslinking mass spectrometry methods, I assume that the peptides were fractionated by size exclusion chromatography in 30% acetonitrile *and* 0.1% TFA. Otherwise you will have retained a lot of peptides by charge.

We thank the reviewer for spotting this, it is correct that both acetonitrile and TFA were present. The methods in page 12 line 6 have been updated accordingly.

2. A table of crosslinked residue pairs along with scores etc should be included with the manuscript.

We have included a table (Supplementary Data 2) with the corresponding crosslinked spectral matches (CSMs) and their corresponding scores, we have only included the inter-protein crosslinked peptides for brevity and relevance to the manuscript.

3. It is best practice to upload mzidentML to PRIDE. This can also be output from XiFDR.

We have submitted a new PRIDE document PXD079187 with the mzidentML files.

4. Crosslinks should be mapped on the Ptdins3K-C1 structure in at least one figure.

We updated Supplementary Figure 3 which now shows all crosslinks between PtdIns3K-C1 and lipidated GABARAP on our cryo-EM structure. Since GABARAP bound to the C site is not seen in this dataset, we represent in this figure only the crosslinked residues on PtdIns3K-C1, and GABARAP is represented as a cartoon in the C site. For the other XL-MS experiment, Figure 5 already shows all crosslinks obtained involving GABARAP, mapped on PtdIns3K-C1 structure.

5. The cryo-EM and HDX-MS descriptions use several site names: NA, NB, N site and C site. These are logical, but the nomenclature becomes dense. A single schematic showing all sites, whether each is visible by cryo-EM, protected by HDX-MS, crosslinked by XL-MS and functionally tested by mutagenesis might help the reader, but is not necessary.

This is an excellent idea, we now added Supplementary Figure 5 to help the readers with the roles of the NA, NB and C sites in our combination of approaches.

6. The model would benefit from a clearer comparison with the previously described WIPI2-PtdIns3K-C1 feedback mechanism motioned.

As we described in the introduction, the WIPI2-PtdIns3K-C1 paper (PMID: 32437499) only demonstrated the WIPI2-PtdIns3K-C1 interaction and did not show whether this interaction affects the PtdIns3K-C1 activity or not. Also, the WIPI2 binding site on PtdIns3K-C1 was not mapped. Therefore, we cannot structurally/biochemically compare the binding/activation mechanisms between mAtg8s and WIPI2. Moreover, as pointed out by reviewer #1, the ULK1 complex and PtdIns3K-C1 are co-recruited to membranes via WIPIs and form a supercomplex, which make difficult the description of the global feedback mechanism when ULK1, PtdIns3K-C1, WIPI2, and GABARAP are all involved in cross-talking positive feedback loops.

7. The authors should define more explicitly whether the increased WIPI2/FIP200 ratio or puncta intensity is being used as a proxy for PtdIns3P production, PtdIns3P persistence, or recruitment efficiency. These are related but not identical.

The WIPI2 puncta intensity corresponds here to total PI(3)P levels, as we could not discriminate between PI(3)P production, persistence or recruitment efficiency of WIPI2 because of the presence of numerous phosphoinositides phosphatases in cells. The text was modified (page 2 line 51) to make this point clearer.

Reviewer #4 (Remarks to the Author):

I co-reviewed this manuscript with one of the reviewers who provided the listed reports. This is part of the Nature Communications initiative to facilitate training in peer review and to provide appropriate recognition for Early Career Researchers who co-review manuscripts.
